# Cancer survivors’ self-efficacy to self-manage in the year following primary treatment

**DOI:** 10.1007/s11764-014-0384-0

**Published:** 2014-07-16

**Authors:** C. Foster, M. Breckons, P. Cotterell, D. Barbosa, L. Calman, J. Corner, D. Fenlon, R. Foster, C. Grimmett, A. Richardson, P. W. Smith

**Affiliations:** 1 Macmillan Survivorship Research Group, Faculty of Health Sciences, University of Southampton, Highfield Campus, Southampton, SO17 1BJ UK; 2 Institute of Health & Society, Newcastle University, Newcastle upon Tyne, UK; 3 Sussex Community NHS Trust, Brighton, UK; 4 Social Statistics & Demography, Social Sciences, University of Southampton, Southampton, UK; 5 Faculty of Health Sciences, University of Southampton, Southampton, UK; 6 University Hospital Southampton NHS Foundation Trust, Southampton, UK

**Keywords:** Self-management, Cancer survivors, Self-efficacy, Confidence, Neoplasms

## Abstract

**Purpose:**

Cancer survivors are increasingly expected to manage the consequences of cancer and its treatment for themselves. There is evidence that self-efficacy is important for successful self-management and that this can be enhanced with support. The purpose of this study was to assess self-efficacy to manage problems in the year following primary treatment.

**Methods:**

This cross-sectional online survey included cancer survivors who had completed their treatment within the past 12 months. Self-efficacy was assessed and variables expected to be associated with self-efficacy were measured using validated scales including quality of life, well-being, illness perceptions, depression and social support.

**Results:**

One hundred eighty-two respondents (mean age 50; 81 % female) completed the survey. They had been treated for a range of cancers; most commonly breast (45 %). Self-efficacy scores varied between individuals and according to the illness-related task to be managed. Respondents were least confident in managing fatigue and most confident in accessing information about their cancer. Individuals most likely to report low self-efficacy were women, those experiencing higher levels of pain and/or depression, lower well-being scores, lower socio-economic status, low levels of social support, or a more negative perception of cancer.

**Conclusions:**

Self-efficacy to self-manage problems faced as a consequence of cancer and its treatment can vary widely in the year following treatment. Fatigue may be particularly difficult to manage.

**Implications for Cancer Survivors:**

Variations in self-efficacy highlight the importance of assessing specific problems faced and people’s confidence to manage them in order to tailor appropriate self-management support.

## Introduction

By 2050, an estimated 70 million people will be living with a diagnosis of cancer worldwide; an almost three-fold increase since 2002 [1]. Rising survival rates are due to improvements in detection and treatments, with many people faring well. However, cancer and its treatment can have a considerable and long-term impact on everyday life [2-4]. With an ageing population and an aftercare system that is not meeting people’s needs, there is growing concern about how best to support cancer survivors and new models of aftercare are being developed and evaluated [5-7].

Self-management in cancer survivorship has been defined as “awareness and active participation by the person in their recovery, recuperation and rehabilitation, to minimise the consequences of treatment, and promote survival, health and well-being” [6]. This will involve managing consequences of cancer and its treatment (physical, psychological, social, practical problems), understanding how and when to seek support, recognising and reporting signs and symptoms of possible disease progression and making lifestyle changes to promote health, well-being and survival. People will be supported to self-manage in a variety of ways but the onus will be on them to initiate contact with healthcare professionals and others to support them [6]. We do not yet understand how able people feel to self-manage. Failure to provide appropriate support could have serious consequences with individuals becoming overburdened, leading to less self-management, greater inequalities, reduced access to services and poorer health and well-being [8].

**Fig. 1:**
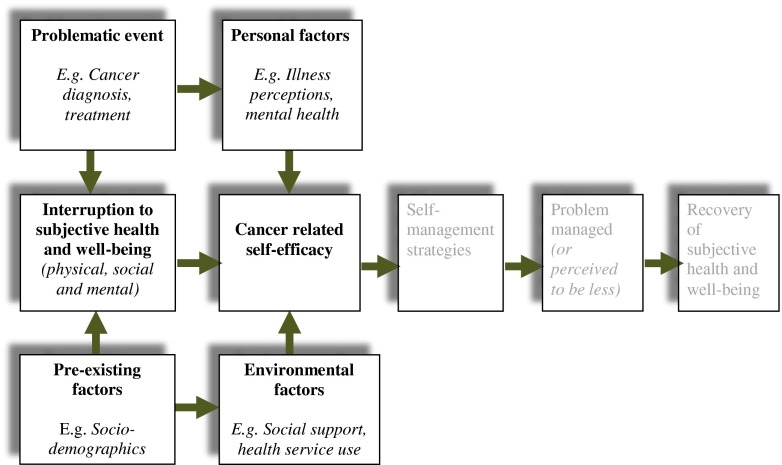
Recovery of health and well-being in cancer survivorship [11]

Self-management can empower cancer patients, increase their confidence to manage problems associated with the disease and its treatment and enhance quality of life [9, 10]. Foster and Fenlon [11] have set out a framework for recovery of health and well-being in cancer survivorship which has self-management and support for self-management as essential components. One element of the framework is cancer-related self-efficacy; belief that one can successfully execute behaviour required to produce expected outcome [12] in relation to consequences of cancer and its treatment. An adapted version of the framework is illustrated in Fig. 1 to reflect elements assessed in this study and demonstrating that the focus of the study is on the factors predicted to be associated with cancer-related self-efficacy. Higher self-efficacy has been associated with a greater effort and persistence to cope with obstacles [13] and enhanced well-being [14]. In people affected by cancer, a high degree of self-efficacy is associated with increased self-care behaviours and decreased physical and psychological symptoms [15]. Self-efficacy is likely to change according to the task to be self-managed and is subject to change [16, 17]. It has therefore become the target of many self-management interventions [10]. Self-efficacy is not a general trait and therefore a person cannot be described as having high self-efficacy or low self-efficacy in all situations. Rather, individuals have beliefs about their ability to carry out tasks and these will vary according to the context and the nature of the task. For example, someone may have high self-efficacy in the workplace but low self-efficacy in relation to exercise. Going further, someone who reports high self-efficacy in the work place may have quite different self-efficacy beliefs when work-related self-efficacy is examined in more detail e.g. high self-efficacy for managing a team but low self-efficacy for delivering a pitch to an audience. For this reason, we have explored cancer-related self-efficacy generally and then looked at different aspects of this to understand areas where cancer survivors may have lower self-efficacy to inform targeted intervention.

Little is known about cancer survivors’ cancer-related self-efficacy and how problems are managed in the year following treatment. This study set out to address this gap. Variables were assessed that were expected to be associated with self-efficacy: cancer and treatment related factors, socio-demographic factors, quality of life, well-being, illness perceptions, depression and social support [11]. Our hypotheses were as follows:

1. Cancer-related self-efficacy will be influenced by socio-demographic and clinical variables
2. Cancer-related self-efficacy will be positively associated with well-being
3. Cancer-related self-efficacy will be negatively associated with depression
4. Cancer-related self-efficacy will be negatively associated with a more threatening perception of illness
5. Cancer-related self-efficacy will be positively associated with social support

The purpose of the study was to identify who is most likely to need support to self-manage cancer-related problems in the year following primary treatment as indicated by lower self-efficacy.

## Methods

### Design

This was a cross-sectional, online survey of adults who had completed primary cancer treatment in the past year.

### Participants

Eligible participants were as follows: ≥18 years; able to complete the survey online in English; had completed primary cancer treatment in the past 12 months; and were resident in the UK. A sample size calculation was performed based on expected variations in the Self-Efficacy for Managing Chronic Disease Scale [18]. Previous studies using this measure have indicated wide variations in self-efficacy scores [19, 20], therefore, the calculation allowed for a 40 % difference between scores; assumed a statistical significance level of 0.05; and a test with 85 % power, giving a required sample size of 170.

### Procedure

Ethical approval was granted by the University (REF: FoHS-ETHICS-2011-051). The survey was created and managed using iSurvey [21]. Recruitment took place August–December 2011. The survey was advertised and linked to 35 charity websites (including social media links), University website, staff bulletins and posters in 260 libraries. Twitter and Facebook accounts were created. Advertisements directed individuals to the study website. Before completing the survey, participants confirmed they had read the Patient Information Sheet and met the inclusion criteria.

### Measures

Validated measures, previously used in cancer populations and informed by our recovery framework were included [11]. Participants provided socio-demographic data and reported health service use.

#### Primary outcome

Self-Efficacy for Managing Chronic Disease Scale was used [18] to measure self-efficacy in people with chronic conditions and has been used with cancer patients [22]. Respondents rate their confidence to perform six self-management behaviours (1 = ‘not at all confident’ to 10 = ‘totally confident’). A mean score is calculated (range 1 to 10). A high score indicates high self-efficacy. We added five cancer-specific self-management behaviours using the same rating scale and calculated a mean score (range 1–10). The new Cancer Survivors Self-efficacy Scale of 11 items was tested using non-parametric, item-response theory (Mokken Scale analysis) [23]. The full set of 11 items formed a strongly homogenous, unidimensional scale of self-efficacy (H = 0.54) with excellent reliability (Alpha = 0.92). We report the mean scale score and also look at individual items to understand the range of self-efficacy beliefs for different aspects of cancer-related self-efficacy.

#### Health and well-being

The Quality of Life in Adult Cancer Survivors scale (QLACS) has been validated with long-term cancer survivors [24]. The scale consists of 12 domains (generic and cancer-specific). Respondents indicate the frequency with which they experienced each quality of life issue in the last 2 weeks (1 = ‘never’ to 7 = ‘always’). Each domain is scored (range 4–28). Higher scores represent poorer quality of life.

The Personal Wellbeing Index (PWI-A) measures subjective quality of life amongst adults. Respondents indicate their global satisfaction with life and satisfaction with eight different aspects of their life (0 = ‘completely dissatisfied’ to 10 = ‘completely satisfied’) [25]. A mean score is calculated and converted into a percentage. A higher value indicates greater sense of well-being. The PWI has been used to assess quality of life in people with spinal cord injury [26], older people [27] and random samples, with good validity and reliability [28].

#### Personal factors

The Brief Illness Perception Questionnaire (Brief IPQ) is an eight-item scale assessing cognitive and emotional representations of illness [29]. Each item is scored on a scale of 0 to 10 (e.g. 0 = ‘no affect at all’ to 10 = ‘severely affects my life’). A total score (after appropriate reverse scoring; range 0 to 80) represents the degree to which the illness is perceived as threatening or benign (Broadbent, personal communication). Higher scores reflect a more threatening view of illness. The instrument has been used with people with cancer [30].

The Center for Epidemiologic Studies Depression Scale (CES-D) assesses how often (0 = ‘rarely or none of the time’ to 3 = ‘all of the time’) participants experienced a range of symptoms in the past week and yields a total score (after appropriate reverse scoring; range 0 to 60) [31]. A higher score indicates more frequent depressive symptoms. A score ≥16 suggests psychological distress. A higher clinical cut-off of ≥20 or 27 has been suggested for cancer patients; although specificity may be reduced i.e. the number of people who are incorrectly identified as NOT having minor or major depression is higher [32]. The CES-D has been established as reliable and valid for measuring depressive symptoms in people with cancer [33].

#### Environmental factors

The Medical Outcomes Study (MOS) social support survey measures availability of social support [34]. Respondents indicate how often different kinds of support are available to them (1 = ‘none of the time’ to 5 = ‘all of the time’). There are 18 items in 4 subscales. A mean score is calculated to produce an overall support index: a higher score denotes a greater level of social support. To compare to published means, scale scores were transformed to a 0–100 scale. This survey has been tested for validity and reliability with cancer survivors [35].

### Analyses

The level of missing data was extremely low. In cases where data contributing to a validated measure were missing, the instructions given by the authors of the measure for the treatment of missing data were followed. Where no explicit instructions for missing data were given, measures based on the sum of scores were not calculated if any items were missing and measures based on a mean score were not calculated if there were more than two items missing. Independent *t* tests, one-way analyses of variance, Pearson and Spearman rank correlations were performed to establish relationships between self-efficacy and other variables. A backward elimination method was used to perform linear regression analysis. Separate regressions, with variables grouped according to the conceptual framework and significant variables, were carried forward to a final analysis. Collinearity diagnostics for all regression models were run; a threshold of <0.1 for tolerance and >10 for the variance inflation factor was observed [36, 37].

## Results

Two hundred thirty-four people completed the online survey. One hundred eighty-two met the eligibility criteria and were included in the analyses; 52 were ineligible (50 had not completed treatment in past 12 months; 2 entered no data).

### Characteristics of the sample

Socio-demographic and clinical data are presented in Table 1. Mean time since treatment completion was 5.36 months. Most respondents were female and described themselves as White British. Ages ranged from 23 to 79 years. Respondents reported 21 different cancer diagnoses. The largest single group were breast cancer survivors. One hundred seventy-seven (97 %) respondents had received surgery, radiotherapy or chemotherapy (or a combination). The most common treatment was surgery. Comorbidities, such as asthma, diabetes and arthritis, were reported by almost 40 % of respondents.

**Table 1:** Socio-demographic and clinical characteristics of respondents

Characteristic	Number or mean (%) *n* = 182
Age	50 years (SD 9.505)
Gender	Female: 147 (80.8)
	Male: 35 (19.2)
Ethnicity	
White British	166 (91.2)
White Irish	5 (2.7)
Other White background	9 (4.9)
White and Hispanic	1 (0.5)
Other mixed background	1 (0.5)
Highest educational attainment	
Higher degree	33 (18.1)
Degree	56 (30.8)
A levels or equivalent	46 (19.8)
GCSE/O levels or equivalent	32 (23.1)
No formal qualifications	15 (8.2)
Domestic status	
Married	103 (56.6)
Divorced/separated	27 (14.8)
Living with partner	22 (12.1)
Single	25 (13.7)
Widowed	3 (1.6)
Missing	2
Employment status	
Employed	132 (72.5)
Not employed	50 (27.5)
Months since completion of treatment	5.36 (SD 3.848)
Treatment received in past 12 months	
Surgery	119 (65.4)
Radiotherapy	102 (56.0)
Chemotherapy	99 (54.4)
Cancer type	
Breast	82 (45.1)
Urological	23 (12.6)
Gynaecological	21 (11.5)
Gastrointestinal	18 (10.0)
Haematological	17 (9.3)
Other	13 (7.1)
Missing	8
Comorbidities reported	70 (38.5)

### Health service use

All respondents had used health services at least once in the past year: 174 (96 %) had seen a General Practitioner, 159 (87 %) an oncologist, 153 (84 %) a cancer nurse and 149 (82 %) a surgeon. One hundred thirty-one (72 %) had stayed overnight in hospital.

### Self-efficacy to manage consequences of cancer and its treatment

**Fig. 2:**
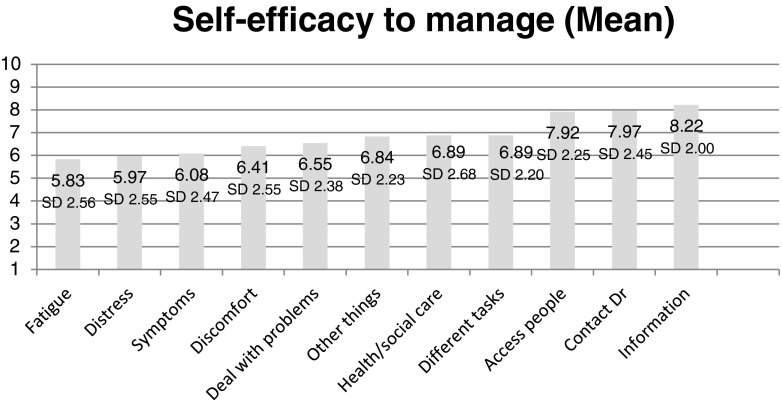
Self-efficacy to manage consequences of cancer and its treatment. Measured by the Cancer Survivors Self-Efficacy Scale (high score denotes higher self-efficacy)

The mean score on the Cancer Survivors Self-Efficacy scale was 6.87 (SD 1.79)^1^ (range: 1–10; higher score = greater self-efficacy). The mean score for each individual item was calculated to highlight variations in self-efficacy across tasks. Self-efficacy to manage fatigue was lowest and self-efficacy to access information about cancer and treatment effects was highest as shown in Fig. 2.

1. Cancer-related self-efficacy will be influenced by the socio-demographic and clinical variables

Some socio-demographic variables were associated with self-efficacy. Male respondents reported higher self-efficacy than females (*t* = 2.595, *p* < 0.05); those who believed their cancer affected the amount or kind of work that they could do were more likely to have lower self-efficacy than those who did not (*t* = 5.857, *p* < 0.01); home owners or those renting from a private landlord reported higher self-efficacy than those renting from a Council or Housing Association or living in temporary or other accommodation (*t* = −2.608, *p* < 0.05). Contrary to Hypothesis 1, cancer-related self-efficacy did not differ significantly by age, marital, or employment status; or clinical characteristics: type of cancer or treatment, time since diagnosis or comorbidities.

### Health and well-being

Hypothesis 2 Cancer-related self-efficacy will be positively associated with well-being

The Generic Summary Score for QLACS was 109.20 (SD 22.68) and the Cancer Specific Summary was 57.20 (SD 19.57). QLACS domain scores ranged from 10.97 (Distress-Family), the least frequently experienced problem, to 18.28 (Energy/Fatigue), the most frequently experienced problem (Table 2). 67 % of respondents reported that they frequently, very often or always experienced problems with fatigue (score ≥5). In terms of concerns about recurrence (scores ≥ 5; frequently, very often or always), 50 % of respondents reported worrying about dying from cancer, 67 % reported worry about recurrence, 53 % reported worrying that pains were a sign of recurrence and 47 % reported being preoccupied with concerns about cancer. Mean PWI was 58.7 (SD 21.8) where a lower score indicates poorer well-being. The normative range for Western populations is 70–80 [36]. There were strong correlations between self-efficacy and QLACS (GSS *r* = −0.65, *p* < 0.001; CSS *r* = −0.52, *p* < 0.001) and PWI (*r* = 0.746, *p* < 0.001). Better health and well-being was associated with higher self-efficacy across all domains (*p* < 0.001) confirming Hypothesis 2.

**Table 2:** Quality of life, illness perceptions, depressive symptoms and perceived social support

Measure	*n*	Mean (SD)
Quality of Life in Adult Cancer Survivors (scores: 4–28; high score = more problems)		
Energy/Fatigue	179	18.28 (4.94)
Distress-Recurrence	182	18.19 (6.53)
Positive feelings	181	16.94 (5.13)
Benefits	182	16.63 (6.12)
Sexual Problems	175	16.26 (6.64)
Pain	181	15.05 (5.79)
Negative feelings	179	15.02 (5.14)
Cognitive Problems	179	14.59 (5.68)
Appearance	180	14.28 (6.93)
Financial Problems	178	13.67 (7.46)
Social Avoidance	175	13.53 (6.44)
Distress-Family	182	10.97 (5.45)
QLACS Generic Summary Score (GSS range 59–161)	168	109.20 (22.68)
QLACS Cancer Specific Summary (CSS range 18–101)	176	57.20 (19.57)
Personal Wellbeing Index (scores: 0–100; high score = better wellbeing)	181	58.7 (21.8)
Brief Illness Perception Questionnaire (scores: 0–80; high score = more threatening perception)	179	39.93 (13.70)
MOS social support survey (scores: 0–100; high score = more support)		
Overall support index	174	67.06 (27.6)
Emotional/informational support	179	61.10 (31.19)
Tangible support	178	66.92 (33.49)
Affectionate support	177	75.99 (32.06)
Positive social interaction	179	73.09 (29.51)

### Personal factors

Hypothesis 3 Cancer-related self-efficacy will be negatively associated with depression
Hypothesis 4 Cancer-related self-efficacy will be negatively associated with a more threatening perception of illness

Mean CES-D was 21.04 (SD 12.44; range 0–60). Of the patients included, 62 % had scores of ≥16, suggesting clinically significant psychological distress; 49 % had scores ≥20 and 32 % scores ≥27. It is unclear what an appropriate cut-off should be, therefore we examined individual items. Ten percent reported that they ‘felt depressed’ ‘all of the time’, 20 % that they ‘felt depressed’ ‘occasionally/moderate amount of the time’. Twenty-five percent reported that they ‘rarely’ felt hopeful for the future. Sixty-six percent of respondents reported restless sleep (‘all of the time’—40 %; or ‘occasionally/moderate amount of the time’—26 %) which may explain the high total CES-D scores. Higher CES-D scores were associated with lower levels of self-efficacy (*r* = −0.75, *p* < 0.001) confirming Hypothesis 3.

Mean Brief IPQ was 39.93 (SD 13.70; range 0–80) where a higher score indicates more threatening perception of the illness. A more threatening perception of illness was associated with poorer self-efficacy (*r* = −0.41, *p* < 0.001) confirming Hypothesis 4.

### Environmental factors

Hypothesis 5 Cancer-related self-efficacy will be positively associated with social support

The mean overall support score was 67.07 (SD 27.6). Least support was reported for emotional/informational support and most support for positive social interaction. However, there is wide variation in the scores for overall social support and the sub-scale scores. Most participants reported adequate social support (score ≥ 4; *most* to *all of the time*) in terms of positive social interaction (65.3 %), affectionate (69 %) and tangible (53.2 %) support. Only 40 % reported adequate emotional/informational support (e.g. someone to confide in, someone to give good advice and information). Higher levels of social support were associated with higher self-efficacy (*r* = 0.53, *p* < 0.001) confirming Hypothesis 5.

### Regression analysis

We conducted a backward, stepwise regression analysis to investigate relationships between self-efficacy and the variables proposed in the selected elements of our conceptual framework as illustrated in Fig. 1 [11]. The final model for self-efficacy (Table 3) included the following: QLACS pain sub-scale, PWI, CES-D, accommodation (owner occupier/renting privately versus not), gender, illness perception and social support. The model accounts for 76 % of the variance in self-efficacy. Variables most strongly associated with a low self-efficacy score were the following: higher level of pain; lower subjective sense of well-being; higher depression; not living in owner-occupied or privately rented accommodation; being female; having a more threatening perception of cancer; having a lower level of social support.

**Table 3:** Regression analysis of factors associated with self-efficacy (*n* = 182)

Model	Unstandardised Coefficients		Standardised Coefficients	95 % Confidence interval for B	
	B	Standard error	Beta		
Constant	7.561	0.614		6.348	8.773
Pain (QLACS)	−0.052	0.015	−0.167**	−0.082	−0.022
Personal wellbeing (PWI)	0.199	0.052	0.240**	0.096	0.301
Depression (CES-D)	−0.029	0.009	−0.200**	−0.047	−0.011
Housing	0.414	0.206	0.081*	0.007	0.821
Gender	−0.415	0.188	−0.090*	−0.785	−0.044
Illness perception (IPQ)	−0.038	0.008	−0.284**	−0.053	−0.022
Social support (MOS)	0.282	0.081	0.171**	0.123	0.442

*R* ^2^ = 0.758

*p < 0.05; **p < 0.01

## Discussion

Compared to studies of cancer survivors, participants in this online survey were doing less well on almost all quality of life dimensions [24]. However, participants in this study had completed their primary treatment more recently (in the past 12 months vs ≥5 years ago). They also reported higher rates of depression, as measured by the CES-D, than reported elsewhere, for example, in head and neck cancer patients up to 6 weeks post radiotherapy [32] and breast cancer survivors 1–5 years post diagnosis [38]. The numbers remained high for clinically significant depression when we raised the clinical cut-off as suggested in the literature for individuals with cancer [32]. Respondents reported greater frequency of fatigue (67 % reporting *frequently* to *always*) than previously reported amongst cancer survivors [5]. The high scores may in part be explained by high fatigue levels and problems with sleep reported by the participants rather than their perceptions of ‘feeling depressed’ so this needs to be interpreted with caution. In relation to personal well-being, participants were doing less well than the general population [28]. Participants in this study had comparable social support scores to others with chronic conditions [34]. There was wide variation in self-reported social support in this sample across the four domains of social support.

Confidence to self-manage problems faced as a consequence of cancer and its treatment varied widely in the 12 months following treatment. Our results are comparable with a study of patients with chronic conditions attending general practices in Germany [39]. Given the higher than expected level of unmet need in our group—particularly in relation to fatigue—it is important to highlight that participants reported low self-efficacy to manage the consequences of fatigue in their everyday lives. Lowest self-efficacy scores were for managing fatigue, emotional distress and health problems.

Relatively high self-efficacy scores for accessing information and support suggests a sample comfortable with accessing information and engaging with support services. This may reflect the relatively high levels of education the sample reported, compared to the general population, as well as a relatively high level of computer literacy implied by participating in the online survey. We have established a clinical cohort of colorectal cancer patients and plan to explore these issues with the cohort participants [40].

As indicated above, levels of self-efficacy varied according to the task, supporting the view that self-efficacy is domain specific [16, 17]. This underlines the importance of considering variation in an individual’s self-efficacy for self-managing different problems associated with cancer and its treatment, rather than viewing individuals as having confidence or not in a general sense. Domain-specific cancer-related self-efficacy measures are likely to be valuable in helping to identify cancer survivors who lack self-efficacy for particular tasks or behaviours. Identification of low self-efficacy in specific behaviours could facilitate targeted support for cancer survivors to better self-manage consequences of cancer and treatment.

Those most at risk of overall low self-efficacy to manage the consequences of their cancer and its treatment were women, those experiencing higher levels of pain, depression, lower wellbeing scores, who did not own their own home or live in privately rented accommodation, had low levels of social support and a more threatening perception of their cancer. Our conceptual model of recovery [11] predicts which factors will impact an individual’s cancer-related self-efficacy. In the model, subjective health and well-being, environmental factors and personal factors influence cancer-related self-efficacy which, in turn, has an effect on an individual’s choice of self-management strategies. These study findings lend support to this model, although as these data are from a cross-sectional survey, we cannot determine causality. Our results, therefore, highlight the possibility of targeting support to cancer survivors who are likely to have lower self-efficacy to manage cancer-related consequences so that they can be supported to self-manage.

Some limitations of this study should be considered when interpreting the findings. Analysis of respondents’ socio-demographic characteristics suggests that these cancer survivors were not representative of the wider population of people living with and beyond cancer. Those taking part in this study were self-selected, younger and more likely to be female, White British and achieved a higher level of education than might be expected in a representative sample of cancer survivors [41, 42]. In addition, the cancer survivors who responded to this survey reported higher levels of psychological distress than might have been expected. In a cancer population, about 20 % would be expected to have clinically significant levels of distress [33]. We have established a prospective clinical cohort of colorectal cancer survivors and will explore cancer-related self-efficacy further in this group [40].

## Conclusions

This study demonstrates that cancer survivors have varying levels of self-efficacy according to different tasks to be managed in the year following treatment. This has implications for how self-efficacy is assessed to identify specific areas of low confidence amongst patients living with cancer or treatment related problems. The regression model indicates a number of factors which may help identify patients at risk of having low confidence to manage problems associated with cancer.

Given that growing numbers of cancer survivors will be expected to self-manage their aftercare, it is important to assess confidence to self-manage the different elements of aftercare, for example, managing consequences of cancer and its treatment; accessing information and support as required; identifying signs and symptoms of possible disease progression; and making lifestyle changes as appropriate. This has implications for targeting support designed to improve self-efficacy.

## Footnotes

1 Mean score for the six items of the original Self-Efficacy for Managing Chronic Disease 6-item Scale was 6.34 (SD 2.05)
